# A Clinical Update on the Prognostic Effect of microRNA Biomarkers for Survival Outcome in Nasopharyngeal Carcinoma: A Systematic Review and Meta-Analysis

**DOI:** 10.3390/cancers13174369

**Published:** 2021-08-29

**Authors:** Peter Shaw, Raghul Senthilnathan, Sunil Krishnan, Deepa Suresh, Sameep Shetty, Gothandam Kodiveri Muthukaliannan, Ravishankar Ram Mani, Palanisamy Sivanandy, Harish Chinna Konda Chandramoorthy, Madan Mohan Gupta, Siddhartha Baxi, Rama Jayaraj

**Affiliations:** 1 Department of Artificial Intelligence, Nanjing University of Information Science and Technology (NUIST), Nanjing 210044, China; 100001@nuist.edu.cn; 2 School of Biosciences and Technology, Vellore Institute of Technology (VIT), Vellore 632014, India; Raghulsnn@gmail.com (R.S.); gothandam@gmail.com (G.K.M.); 3 Department of Radiation Oncology, Mayo Clinic Florida, Jacksonville, FL 32224, USA; Krishnan.Sunil@mayo.edu; 4 Division of Endocrinology, Department of Internal Medicine, Mayo Clinic Florida, Jacksonville, FL 32224, USA; Deepa.Suresh@mayo.edu; 5 Department of Oral and Maxillofacial Surgery, Manipal College of Dental Sciences, Manipal Academy of Higher Education, A Constituent of MAHE, Manipal 576104, India; sameep.shetty@manipal.edu; 6 Department of Pharmaceutical Biology, Faculty of Pharmaceutical Sciences, UCSI University, Cheras, Kuala Lumpur 56000, Malaysia; Ravishankar@ucsiuniversity.edu.my; 7 Department of Pharmacy Practice, School of Pharmacy, International Medical University, Bukit Jalil, Kuala Lumpur 57000, Malaysia; PalanisamySivanandy@imu.edu.my; 8 School of Postgraduate Studies, International Medical University, Bukit Jalil, Kuala Lumpur 57000, Malaysia; 9 Department of Microbiology and Clinical Parasitology, College of Medicine, King Khalid University, Abha 61421, Saudi Arabia; hshkonda@kku.edu.sa; 10 School of Pharmacy, Faculty of Medical Sciences, The University of the West Indies, St. Augustine 3303, Trinidad and Tobago; madanmohan.gupta@sta.uwi.edu; 11 John Flynn Hospital, 42 Inland Drive, Tugun, QLD 4224, Australia; Siddhartha.baxi@genesiscare.com; 12 Northern Territory Institute of Research and Training, Darwin, NT 0909, Australia

**Keywords:** nasopharyngeal carcinoma, miRNAs, biomarkers, prognosis, survival, systematic review, meta-analysis

## Abstract

**Simple Summary:**

Current estimates by GLOBOCAN now incorporate NPC as a malignancy discrete from other head and neck malignancies among the 36 disease locales assessed. Based on the latest report, the global cancer burden is estimated to have risen to 19.3 million new cases, and 9.6 million malignancies were recorded in 2020 throughout the world. The study has clinical implications and could improve treatment decision-making and post-treatment care. The study could also motivate future clinical research and development in the arena of NPC prognostic biomarkers.ve men and one in every six women develops cancer during their lifetime, and one out of eight men and one in every 11 women progresses to chronic stage. The study has clinical implications and could improve treatment decision-making and post-treatment care. The study could also motivate future clinical research and development in the arena of NPC prognostic biomarkers.

**Abstract:**

*Background*: Nasopharyngeal carcinoma (NPC), a relatively uncommon malignancy in the Western world, is highly prevalent in Southeast Asia where the treatment outcomes are poor. Despite recent improvements in diagnosis and treatment locoregional control, distant metastasis and chemoresistance continue to be a significant cause of mortality. Identification of a reliable and comprehensive prognostic biomarker is highly desirable. The potential relevance of microRNAs (miRNAs) as prognostic markers in NPC is assessed in this systematic review and meta-analysis. *Methods*: A systematic review was performed using the PubMed and Science Direct databases. The search was limited to search results between 2018 and 2020 with the keywords and search strings developed as per the Preferred Reporting Items for Systematic Review and Meta-analysis (PRISMA) guidelines. The recovered articles were carefully screened based on the selection criteria. In the meta-analysis study, high and low expression levels of miRNAs were measured using the hazard ratio (HR) and 95 percent confidence interval (CI) for patients’ survival outcomes. Egger’s bias indicator test and funnel plot symmetry were used to assess the risk of bias. *Results*: Amongst the 25 studies, 13 fulfilled the conditions of inclusion in this meta-analysis. The researchers further delved into the 21 miRNA expression levels from 3015 NPC patients to ascertain a link between miRNA’s predictive role and survival outcomes. The majority of the articles retrieved during this study were from China, with two studies from Canada and Malaysia. The overall pooled effect size estimation (HR) for dysregulated miRNAs was 1.590 (95% CI: 1.253–2.017), displaying that miRNA marker expression increased the risk of mortality in NPC patients by 59%. *Conclusions*: This meta-analysis is novel and looks at the prognostic significance of miRNAs as biomarkers in NPC patients using a continuous version pooled meta-analysis. Although our findings are ambiguous, they do show that greater miRNA expression in NPC may be associated with a lower overall survival rate. To acquire clear conclusions, more prospective studies with large cohorts are required to determine the clinical utility of miRNAs as prognostic biomarkers.

## 1. Introduction

Nasopharyngeal carcinoma (NPC) is a type of epithelial cancer that is distinct from other head and neck cancers. The endemic variation highly prevalent in Southeast Asia has direct links to Epstein-Barr virus (EBV) infection and regularly presents as an undifferentiated histologic subtype [1]. Current estimates by GLOBOCAN now incorporate NPC as a malignancy discrete from other head and neck malignancies among the 36 disease locales assessed [2] According to a 2013 report, NPC was responsible for nearly 42,100 new patients and 21,320 deaths in China alone in said year. Of this total incidence, around 30,000 were new male NPC patients, representing 1.47% of all new male cancer patients and a crude incidence of 4.31/100,000, and around 12,000 were new female cases, representing 0.74% of all new female cancers and a crude incidence of 1.81/100,000 [3]. In view of these reports, Asia registered a total of 133,354 new NPC cancers per year [4], whereas less than one person in every 100,000 people in the United States is diagnosed with NPC. The failure rate after NPC treatment is primarily due to recurrence (5–15%) and distant metastasis (15–30%) [5]. The 5-year survival rate (people who live at least five years after cancer detection) for people diagnosed with NPC is 61%.

Since its inception, miRNAs have been observed to have significant ramifications in carcinogenesis, cancer progression, and response to treatment. miRNA expression markers have been demonstrated to be potential new biomarkers for malignancy detection, prognosis, and treatment response evaluation [6,7,8]. EBV-miRNAs play a substantial role in the pathological process of EBV-driven cancers including NPC, largely via their impact on anti-growth and apoptotic pathways [9,10]. Several miRNA-based biomarkers have been developed to classify different types of cancers into homogeneous groups based on specific miRNA expression signatures. Prostate cancer [11], lung cancer [12], breast cancer [13], skin cancer [14], and head and neck cancer [15] are all common examples for such differential expression. This systematic review and meta-analysis outline the role of miRNA in NPC prognosis and response to treatment.

## 2. Rationale

### 2.1. The Significance of miRNA

MiRNAs have been studied for their prognostic efficacy in a variety of head and neck cancers (HNC), including laryngeal, squamous cell carcinoma and oropharyngeal carcinoma. The prognostic role has been reported and investigated through various narrative reviews, systematic reviews, and meta-analyses [16,17,18]. Previous studies focused primarily on other anatomical subsites of HNC, but there is a lack of knowledge about the prognostic effect of miRNA regarding NPC. Knowledge regarding such prognostic effects could be a valuable tool in improving treatment strategies for head and neck care. The presence of circulatory EBV biomarkers in blood prior and post-treatment has previously been shown to have predictive significance in cancer management [19]. However, these results have not been consistent across studies and patient populations.

Deregulated expression of some miRNAs has been highlighted in a few reviews as being associated with the presence and progression of NPC as well as its prognosis [8,20,21]. As previously reported, downregulation of certain specific miRNAs, has been linked to improved survival in NPC patients [22]. miRNA target prediction and pathway enrichment analysis were utilized in previous meta-analysis to identify and specify the functional genes involved in NPC regulation. However, researchers in this study focused solely on potential miRNA contributions to NPC pathogenesis and signaling pathways, rather than on overall or treatment outcomes [23]. There are abundant systematic reviews, narrative reviews and meta-analysis that have indicated diagnostic, prognostic, and therapeutic utility of miRNA in several subtypes of head and neck cancers, nonetheless, there has not been a quantitative and qualitative evaluation of the use of miRNA in NPC patient survival through a review or meta-analysis [24,25]. The dearth of adequately validated molecular biomarkers that perform as reliable prognostic indicators in NPC and HNC accounts for the observed deficiency.

Previous systematic reviews and meta-analyses focused on biomarkers in NPC that had limited sample size, small numbers of included studies, and scant information on survival. A report on the hematologic markers of prognostic significance in NPC analyzed the patient survival parameters such as overall survival (OS), progression-free survival (PFS), cause-specific survival (CSS), and local relapse-free survival (LRFS) among NPC patients as a function of neutrophil-to-lymphocyte ratio (NLR) and absolute lymphocyte counts and concluded that high NLR was associated with poor NPC while increased lymphocyte counts portended a favourable prognosis. However, this study was limited to East Asian patients [26]. Another study on circulating miRNAs in NPC studied the platelet-to-lymphocyte ratio (PLR), NLR, diagnostic odds ratio (DOR), and summary receiver operating characteristics (SROC) to calculate the overall diagnostic accuracy and summarized the potential of circulating miRNAs. However, this study included only seven systematic reviews and four studies for meta-analysis, making the results less conclusive [27]. The prognostic significance of the epidermal growth factor receptor (EGFR) pathway in NPC was evaluated in a systematic review and meta-analysis and concluded that the overexpression of EGFR predicted worse OS and disease-free survival (DFS) [28]. A study by Liu and colleagues on the genome-wide serum miRNA profiling in NPC using four types of miRNAs (miR-22, miR-572, miR-638, and miR-1234) revealed that the TNM stage and the miRNA involved were independent prognostic factors [7].

### 2.2. How Will the Research Deal with the Problem?

The purpose of this study is to bridge the lacunae about miRNAs’ potential as prognostic biomarkers in NPC. A systematic review and comprehensive meta-analysis were conducted to provide comprehensive evidence for assessing the effect of miRNA on patient survival through identifying the combined effect across the various studies assessing NPC and miRNA. This study summarizes an estimate of the association between rising levels of miRNA expression and the risk of death across all of the studies (using the effect size of the Hazard Ratio) that could help us better understand NPC patient survival. This updates our previous study on the quantitative and qualitative analysis of published research on NPC prognosis [29]. This analysis has the potential to be immensely helpful to clinical researchers and biologists in their understanding of the disease at a molecular level.

### 2.3. What Effect Will It Have?

This research will help ascertain the effect of miRNA expression on NPC patients’ prognosis. It will also enable us to use biomarkers to estimate survival and perform future studies determining the role of miRNAs in predicting disease progression and survival. The study has clinical implications and has the potential to improve treatment decisions and post-treatment care. The study may also serve as a springboard for future on the topic of NPC prognostic biomarkers, clinical research and development is ongoing.

## 3. Methods

The Preferred Reporting Items for Systematic Review and Meta-analysis (PRISMA) criteria were used to perform the study [30]. This study is based on the PROSPERO that was registered under the ID CRD42018083945.

### 3.1. Search Strategy

To ascertain the proposed meta-analysis, the PubMed and Science Direct databases were searched for studies concluded between 2018 and 2020. The search was performed using the Medical Subjective Heading (MeSH) search terms. Our core search consisted of binding terms that included all abbreviations, synonyms, and subsets (Table 1). Two reviewers examined the titles and abstracts independently to determine whether the articles met our inclusion criteria. For studies without abstracts, full-text articles were examined. Any disagreements between the two reviewers were resolved by a third reviewer or consensus-based discussion with the corresponding author.

### 3.2. Selection Criteria

Eligible studies were incorporated based on the following predefined eligibility criteria.

#### 3.2.1. Inclusion Criteria

(1) Research was published from 2018 through 2020.
(2) Platforms for miRNA profiling that have been reported in several studies.
(3) Studies that explored the prognosis of miRNA in NPC patients
(4) Research into the resistance to a particular type of treatment.
(5) The study used clinical patient data.
(6) Studies in which OS, PFS, DFS, distant metastasis-free survival (DMFS), or recurrence-free survival (RFS) were elucidated by Hazard Ratio (HR) and 95 percent confidence intervals (95 percent CI) can be calculated numerically or using Kaplan-Meier curves.
(7) PRISMA standards for systematic review and meta-analysis were followed in these studies.

#### 3.2.2. Exclusion Criteria

(1) Manuscripts written in a language other than English.
(2) Lack of patient survival data.
(3) Studies using duplicated data.
(4) Studies that included non-human data
(5) Unpublished materials, where conference proceedings, incomprehensible data, or theses are all examples of unpublished materials.
(6) Fact sheets, cohort studies, intervention studies, reviews, case-control studies, laboratory investigations, letters to editors, and non-human studies are some of the types of research that are available and uneligible for inclusion.

### 3.3. Data Extraction and Management

All studies that met exclusion and inclusion criteria were evaluated, and all the particulars about patient clinical and histological parameters were retrieved. After a thorough examination of the Author names, year of publication, study location, study period, gender, sample size, source of a clinical sample, miRNAs profiling platform, follow-up period, miRNAs studied, histological type, lymph node metastasis/distant metastasis, clinical stages, and survival data (HR and 95%CI) were all sorted under the following headings: author names, year of publication, study location, study period, gender, sample size, source of a clinical sample, miRNA (including OS, DFS, DMFS, RFS, PFS). All of the data gathered from the studies that qualified for final inclusion was entered into a Microsoft Excel spreadsheet.

## 4. Results

### 4.1. Study Selection

**Figure 1:**
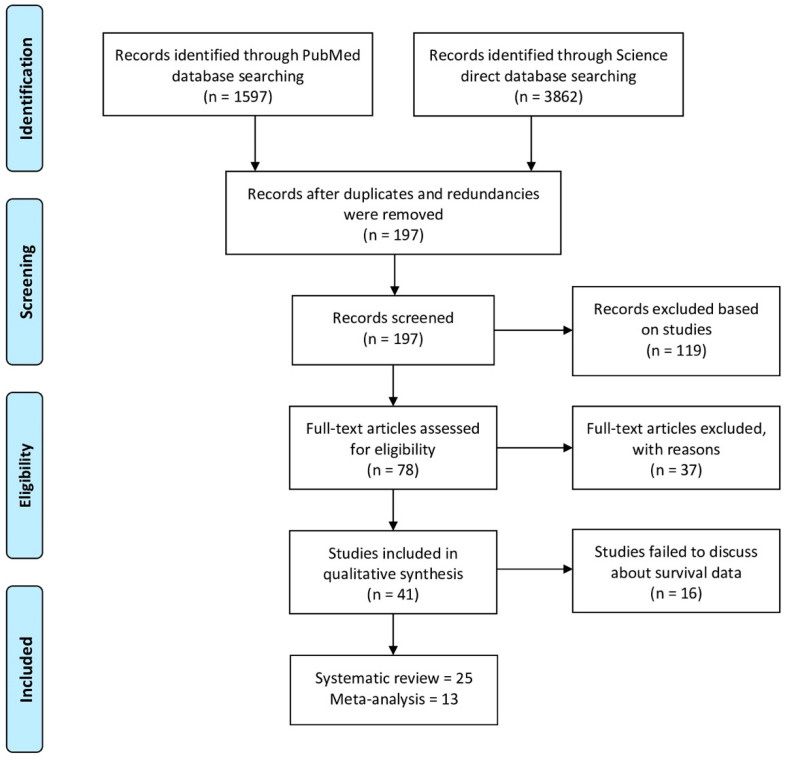
Schematic representation of selection of articles for this meta-analysis and a comprehensive review.

The scheme for selecting articles for final inclusion is depicted in Figure 1. The initial search yielded 5459 articles from the PubMed (*n* = 1597) and Science Direct (*n* = 3862) databases. After removing the ineligible studies based on the exclusion criteria, 197 articles were considered for screening, and out of 197 articles, 119 further records were excluded based on their being reviews, case-control studies, or cohort studies. Accordingly, 78 articles were deemed relevant and were included for further analysis. Following the full-text screening, 37 additional studies were excluded because they were outside the scope of the study, including non-human sample studies (*n* = 11), conference abstracts (*n* = 2), studies whose full-texts were unavailable (*n* = 6), NPC studies that did not assess miRNA marker expression (n = 5), and studies lacking required data (*n* = 13). Upon examining the full-text studies included in qualitative synthesis (*n* = 41) against the inclusion criteria, seven studies did not explore the prognosis of miRNA in NPC patients; four studies did not directly deal with miRNA expressions, five studies did not report survival endpoints in NPC patients. Finally, 25 studies were included in the systematic analysis. Among these, only 13 papers were found to be eligible for extraction of HR with 95% confidence intervals either directly or on the basis of Kaplan-Meier curves.

### 4.2. Study Characteristics

The main characteristics of the study are represented in Table 2. Of the 25 studies, 23 were primarily conducted in China, one in Canada, and one in Malaysia. A total of 3015 patients were compiled for analysis, with cohort sizes ranging from 30 to 558 patients in the individual studies. miRNA expression profile has been analyzed in fresh/preserved tissue samples (20 studies), plasma (two studies), serum (two studies), and saliva (one study). miRNA quantification was performed using qRT-PCR exclusively in 20 studies, while microarray analysis was used in one study and both qRT-PCR and microarray analysis were used in four studies. All studies reporting the status of miRNA dysregulated expression were included in the systematic review and meta-analysis, with the exception of five miRNAs (miR-639, miR-432, miR-495, miR-BART13-3p, and miR-BART13-3p) in four studies [31,32,33,34]. The remaining 55 miRNAs were upregulated in 19 cases and downregulated in 36 cases.

### 4.3. Comprehensive Meta-Analysis

**Figure 2:**
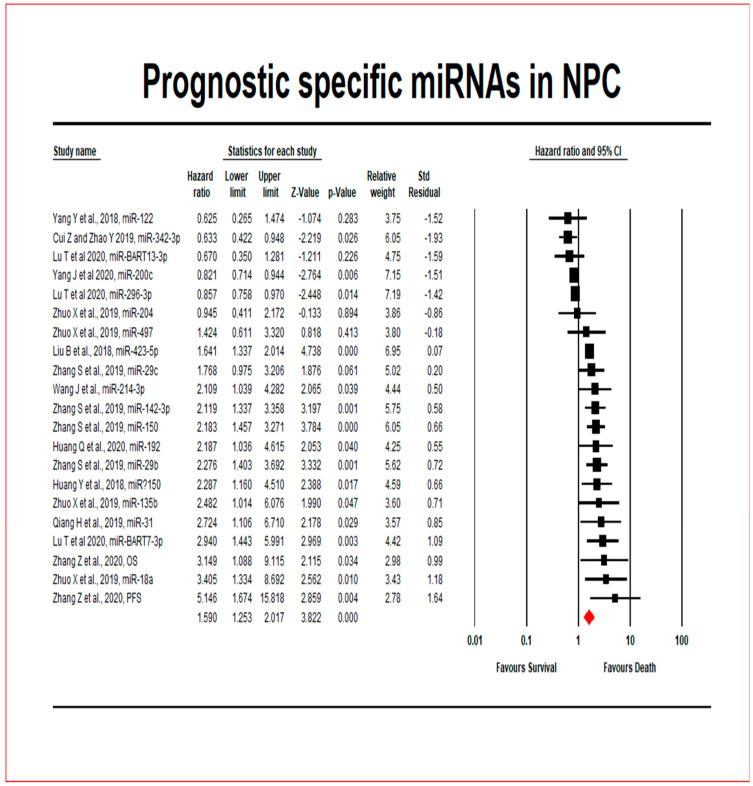
The survival outcome of miRNAs in NPC patients is represented by a forest plot.

In 1116 NPC patients from 13 included studies, the prognostic significance of 21 miRNAs was investigated (Figure 2). Seven miRNAs were upregulated, while 12 miRNAs were downregulated. Dysregulation of two miRNAs was not reported in one study [34]. The overall pooled effect estimates of HR for (upregulated and down-regulated) miRNA expressions were 1.590, with a 95 percent confidence interval of 1.253–2.017, meaning that miRNAs expression increased the risk of mortality in NPC patients by 59 percent when using the random effects model. Table 3 displays the heterogeneity and hypothesis testing.

### 4.4. Does the Expression of miRNAs Influence the Survival of NPC Patients?

The null hypothesis test Z value was 3.8222, and the corresponding *p*-value was 0.000. Indicating that the risk of death was higher in upregulated groups than in downregulated groups. Out of these 21 miRNAs across 13 studies, six miRNAs (miR204, miR122, miR342-3p, miR200c, miR296-3p and miR-BART13-3p) were associated with a better prognosis, while fifteen miRNAs (miR423-5p, miR497, miR31, miR150, miR192, miR214-3p, miR18a, miR135b, miR142-3p, miR150, miR29b, miR29c, miR125b, miR26b, and miR-BART7-3p) indicated a poor survival.

### 4.5. How Much Does the Extent of the Estimated Effect Size of Npc Patients Vary across the Included Studies?

The Q-statistic tests the null hypothesis that all analysis studies have the same impact size. With 20 degrees of freedom (df) and a *p*-value of 0.000, the Q-value was determined to be 130.343. Because the observed variance falls within the range attributed to sampling error, we cannot reject the null hypothesis that the actual effect size was the same in all of the included studies. The I^2^ statistic refers the extent of the observed variance, which indicates differences in exact effect sizes rather than sampling error. I^2^ is 84.656 percent in this case. The variance of accurate effect sizes is denoted by T^2^ (T = tau) (in log units). T^2^ is 0.201 in this study. The standard deviation of actual effects is denoted by T (in log units). T (tau) is 0.448 in this study.

### 4.6. Is There a Difference in the Extent of the Effect Based on the Subgroup of NPC Patients Who Survive? 

While the overall effect size is small (HR 1.590) (Figure 2 and Supplementary Figure S1), it varies by subgroup. We compared the extent of the effect in trials with high and low miRNA expression using subgroup analysis. The differences had a Q-value of 130.343 with 20 df and a p-value of 0.000. As a result, there was no evidence that HR was related to NPC patient survival.

### 4.7. Publication Bias and SensitivityAnalysis – Funnel Plot

**Figure 3:**
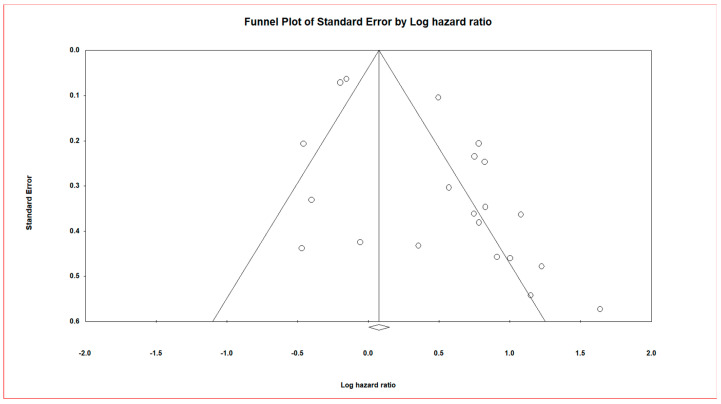
Funnel plot of Standard Error by Log hazard ratio correlating patient survival in general and microRNA expression.

Figure 3 depicts a funnel plot that was slightly asymmetric across survival outcomes. The funnel plot of the overall studies is depicted in Supplementary Figure S2. The asymmetry of the funnel plot suggests the presence of publication bias. The asymmetry could be related to small-study effects (such as sampling error).

CMA software (version 3.3.070, Biostat Inc. Eglewood, NJ, USA) was used to calculate and analyse the HR values’ pooled hazard ratios for NPC prognostic data. The black square in the forest plot with lines is the pooled effect size estimate of survival for NPC patients randomly assigned to miRNA evaluation.The pooled effect estimate suggests that upregulation of miRNAs in NPC leads to poorer overall survival by a magnitude of 1.5 times.

On the vertical axis, the funnel plot shows the study size’s standard error and precision as a function of the effect size on the horizontal axis. Individual studies are shown by dots, and the majority of this area has high-significance regions, indicating the probability of publication bias. Smaller studies (which appear at the bottom) have higher probability of publication if their effects are larger than average.

This meta-analysis included data from 13 NPC studies examining 21 miRNAs that yielded a Z-value of 6.34554 and a corresponding two-tailed *p*-value of 0.00. The fail-safe N test reported that 200 null studies would be needed to reduce the effect to non-significance (two-tailed *p*-value to exceed 0.050). It was observed that we had a significant fail-safe N; from this, we can be confident that the prognostic effects could have been inflated by excluding some studies; nevertheless, it was not nil.

#### 4.6.1. Orwin’s Fail-Safe N Tests

The HR in the observed studies was found to be 1.07586. The mean hazard ratio in the missing studies is 1.000 (it can be a value other than nil value). The criterion value must fall between the other values for the Orwin fail-safe N to be estimated. Here the HR in observed studies, which was found to be 1.07586, did not fall between the mean HR in the missing (new) studies, which is 1.000.

#### 4.6.2. Begg and Mazumdar Rank Correlation Test

The rank-order correlation (Kendell’s tau) values were found to be 0.07619 (without continuity correction and 0.07143 (with continuity correction). The *p*-values for 1-tailed and 2-tailed were determined to be 0.32529 and 0.65058, respectively.

#### 4.6.3. Egger’s Test of the Intercept

In this study the intercept (B0) was found to be 2.43151, 95% CI (1.03940–3.82362), with *t*-value = 3.65575 and df = 19.000. The recommended 1-tailed *p*-value was 0.00084 and 2-tailed *p*-value was 0.00168. The intercept (B0) was determined to be 1.83640, 95% CI (0.78221–2.89059), with a *t*-value of 3.51307 and a df of 43.000 in the pooled studies. The 1-tailed *p*-value should be 0.00058, and the 2-tailed *p*-value should be 0.00106.

#### 4.6.4. Trim and Fill at Duval and Tweedie’s

**Figure 4:**
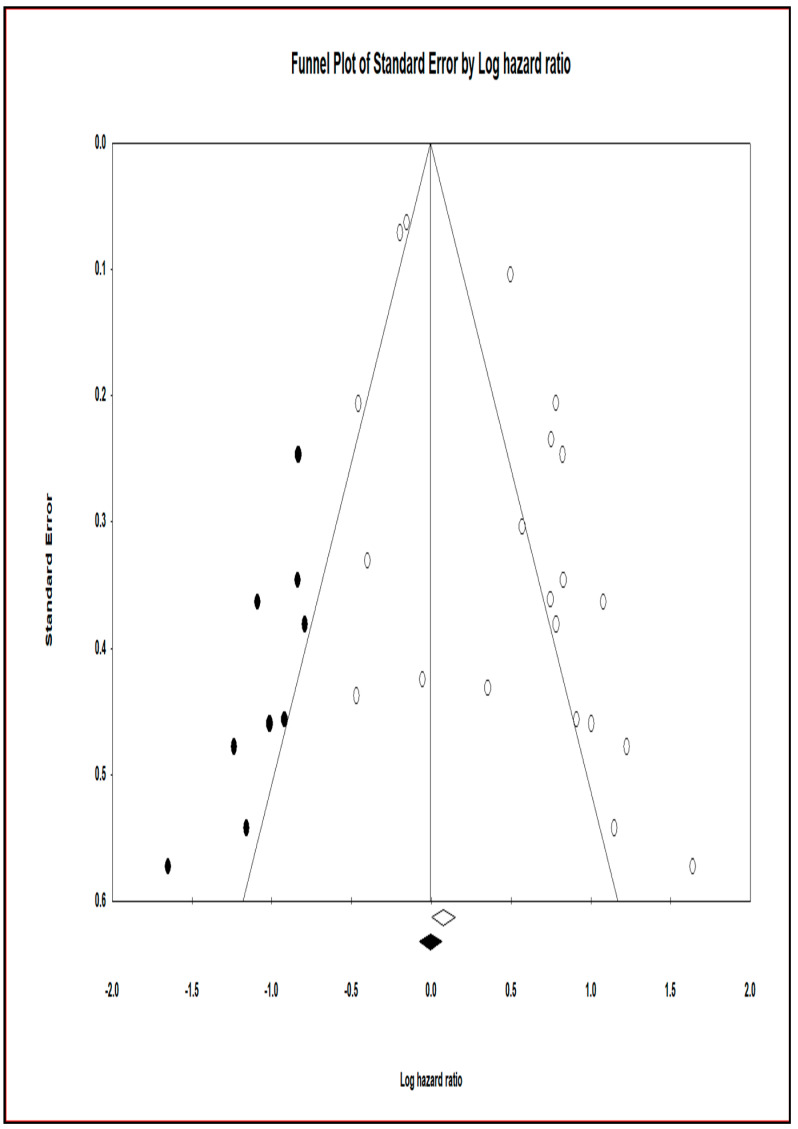
Funnel plot with observed and imputed studies.

To reduce the influence of publication bias, approximately 9 studies that produced the asymmetry in the funnel plot were clipped and filled using this procedure. CMA software was used to create funnel plots for trimmed and imputed studies (Figure 4). The funnel plot with imputed studies pertaining to the total studies is shown in Supplementary Figure S3. The hazard estimate and 95% CI for the current studies were 1.07856 (1.00199–1.16097) and 1.01814 for the combined studies, respectively, using the Fixed effect model (0.96611–1.07297). The adjusted values for the point estimate and 95 percent CI for the current investigations were 0.99430 (0.92641–1.06716) and for the combined studies were 0.93413 (0.92641–1.06716) through Trim and Fill (0.88792–0.98274). The hazard point estimate and 95 percent confidence interval for the current studies were 1.58998 (1.25343–2.01689) and 1.42493 for the combined studies, respectively, using the random effect model (1.19441–1.69993). The corrected values for the point estimate and 95% CI for the new studies were 1.07780 (0.86076–1.34956) and 0.99886 for the combined studies using Trim and Fill (0.83723–1.19170).

The funnel plot plots the standard error and precision of the study size on the vertical axis as a function of the effect size on the horizontal axis. Individual studies are represented by dots, and the majority of this area contains regions of high significance, indicating that publication bias is represented as asymmetry. This would imply that smaller studies (which appear at the bottom) are more likely to be published if they have larger-than-average effects, making them more likely to meet the statistical significance criterion. From the 45 published studies, we focused on the effectiveness of using miRNA as biomarkers in patients suffering from NPC. Different publication bias modules, funnel plots, regression tests, and trim and fill methods helped support our study. Standardization of specific miRNA is an essential requirement in the prognostication of NPC. To assess the reproducibility and the inter-observer variability, we require sizeable prospective observational clinical studies.

## 5. Discussion

Our earlier research observed for a relationship between miRNA expression levels and patient survival in NPC patients [29]. Several evaluations have suggested that miRNA could be used to predict the prognosis of NPC patients, as previously indicated. The primary goal of this meta-analysis and systematic review is to support our previous review and broaden the clinical applicability of this analysis. Our research has also observed how miRNA expression differs depending on factors such as gender and clinicopathological characteristics. Both univariate and multivariate analysis were used to investigate the survival rate of NPC patients. This shows that males are more naturally predisposed to NPC than females [56]. Males are about two to three times more likely than females to develop NPC. This is explained by a number of factors, including male gonadocorticoids, the effect of endogenous estrogen, and a higher prevalence of smoking among men [57]. The rate of NPC occurrence in low-risk groups was similar to that of epithelial cancers and increased with age. The bimodal pattern of incidence as a function of age is highlighted by an initial peak between the ages of 50 and 59, and a subsequent peak in the seventh decade [58]. Literature is replete ascertaining the link between a patient’s age and the occurrence of NPC, but there is scarcity of evidence indicating age has a statistically significant impact on a patient’s survival [59].

Overexpression of miR17-5p was linked with the occurrence and proliferation of tumours by downregulating other proteins like the p21 protein [60]. Zhang et al. reported that miR17-5p in plasma showed a consistent upregulation in NPC patients compared to normal controls [52]. With the data standardized to miR-39 spike-in control, Tan et al., using RT-qPCR results, demonstrated that over 38.9% of miRNAs detected in the nasal wash were significantly upregulated in the NPC pre-treatment when compared with the standard control samples. They also suggested that miR93 and miR205, which were upregulated in NPC, could enhance the cell growth and migration in NPC cell lines [41]. From their study, Zhao et al. proposed that miR20a-5p upregulation in NPC could enhance resistance to radiotherapy for the patients [61]. Let-7d expression was found to be low in HNC patients, which was linked to a poor prognosis. As a result, let-7d inhibits tumorigenesis and promotes apoptosis in tumour cells via AEG-1 [62].

### 5.1. Strengths

The articles included in the systematic review and meta-analysis were newly published studies from all over the world. To update the different published articles on miRNA molecular predictors for NPC, the researchers utilised a comprehensive database search and analytic strategy. Because the selected/included studies were acquired from recently published studies studying miRNA expression and prognosis, they could be used in future NPC studies. The majority of the studies were assessed to be of valid quality, according to the assessment score for the included NPC studies. The drive of this systematic review and meta-analysis is to update miRNA prognostic estimates and their utility as prognostic biomarkers in NPC. Furthermore, all of the research included in the study complied with the PRISMA recommendations. The studies’ methodological quality was demonstrated through the use of quality evaluation methods.

### 5.2. Limitations

The paper’s global clinical applicability may be limited as the included studies were conducted only in three countries (Canada, Malaysia, and China). To compensate for the HR results that were not provided, the HR results and the 95% confidence intervals were evaluated from the Kaplan Meier curves presented graphically, which may induce minor errors in the analysis. Inherent to such analyses are heterogeneity in assay methodologies across studies. The bias arises from the different methods utilized for each study, as carrying out techniques will differ among laboratories due to the different sources of kits and reagents possessed by them, thus leading to a varying threshold level that has an ever-varying value. Subgroup analysis could not be performed because the number of included studies was very small. Due to the inaccessibility of HR and CI data in the included studies, subgroup effects of miRNAs based on (recurrence) variables were also not possible.

## 6. Conclusion

Our update of the systematic review and meta-analysis offers additional support for the hypothesis that miRNAs play a pivotal role in NPC prognosis. The comprehensive meta-analysis expounded on the influence of dysregulated miRNA expression on the survival of NPC patients. Our study suggests that increased miRNA expression is associated with poor overall survival in NPC patients. Besides, it also advocates that researchers should concentrate on large-scale cohort studies to validate the prognostic significance of miRNAs. To further elucidate their applicability in the clinical setting, comprehensive clinical investigations with large sample sizes are essential to benchmark and validate the use of miRNAs as biomarkers and determine their impact on NPC patient survival outcomes.

## Figures and Tables

**Table 1:** Key terms utilized in the search strategy.

1	“Nasopharyngeal cancinoma” [Topic] AND “miRNA” [Topic]
2	“NPC” [Topic] AND “Chemoresistance” [Topic]
3	“Prognosis” [Topic] AND “Chemo resistance” [Topic]
4	“miRNA” [Topic] AND “Biomarkers” [Topic]
5	“miRNA” [Topic] AND “NPC” [Topic] AND “Prognosis” [Topic]
6	“Prognosis” [Topic] OR “Survival outcome in NPC” [Topic]
7	“Upregulation” [Topic] OR “Downregulation in NPC” [Topic]
8	“Follow up studies.” [Topic] OR “miRNA” [Topic]
9	“Systematic review” [Topic]“Meta-analysis study” [Topic] AND “NPC” [Topic]

**Table 2:** The main features of the studies included in the systematic review and meta-analysis.

Study	Population	Study Period	Gender	Sample Size	Source of Sample	Platform	Follow-up Period	miRNA Studied	WHO Histological Type	Lymph Node Metastasis/Distant Metastasis	T Stage	Endpoints	HR Value	miRNA Dysregulation
L Ju et al., 2018 [35]	China	2007 and 2015	M-83/F-27	110	Tissue	qRT-PCR	5 Year	miR-9	NA	N0-N3	T1, T2, T3 & T4	OS	KM Curve alone	Upregulated
He H et al., 2018 [36]	China	March 2013 and November 2014	NA	42	Tissue	qRT-PCR	NA	miR-494-3p	NA	NA	NA	NA	NA	Upregulated
Zhao L et al., 2018 [37]	China-312/Canada-246	January 2003 and February 2006	NA	558	Tissue	qRT-PCR	62.1 Months	miR-29b, miR-29a, and miR-26a	NKUC, NKDC, KSCC	N0-N3	T1, T2, T3 & T4	OS, DFS, DMFS	KM Curve alone	Downregulated
Lian Y et al., 2018 [38]	China	NA	NA	45	Tissue	qRT-PCR/ Microarray analysis	NA	miR-423-5p	NA	NA	NA	OS, RFS	KM Curve alone	Downregulated
Liu B et al., 2018 [39]	China	May 2011 to May 2013	M-37/F-57	94	Serum	qRT-PCR	36 Months	miR-150	NKUC, NKDC, KSCC	Studied but not mentioned exact stage	T1, T2, T3 & T4	OS	KM Curve alone	Upregulated
Wang YH et al., 2018 [31]	China	March 2013 to July 2015	M-94/F-45	139	Tissue	qRT-PCR	NA	miR-639	NKUC, NKDC	NA	NA	DFS	KM Curve alone	NA
Liu Y et al., 2018 [40]	China	NA	M-32/F-9	41	Tissue	qRT-PCR	NA	miR-141	NKUC, NKDC	NA	NA	NA	NA	Upregulated
Wang T et al., 2019 [32]	China	NA	M-47/F-19	66	Tissue	qRT-PCR	NA	miR-432	NA	N0-N3	T1, T2, T3 & T4	NA	NA	NA
Tan GW et al., 2019 [41]	Chinese-68/Malay-44/Others-7	NA	M-88/F-31	119	Tissue	qRT-PCR	NA	miR-21, miR-26a, miR-29c, miR-93, miR-205, miR-375 and miR-421	NA	N0-N3	T1, T2, T3 & T4	NA	NA	miR-21, miR-93, miR-205, and miR-421—Upregulated, miR-26a, miR-29c, and miR-375—Downregulated
Zhuo X et al., 2019 [42]	China	NA	M-11/F-51	62	Tissue	Microarray analysis	NA	miR-18a, miR-135b, miR-204, and miR-497	NA	Studied but not mentioned the exact stage	NA	OS	KM Curve alone	miR-18 and miR-135b—downregulated, miR-204 and miR-497—Upregulated
Qiang H et al., 2019 [43]	China	January 2013 to December 2015	M-32/F-24	56	Tissue	qRT-PCR	NA	miR-31	KSCC	NA	NA	OS	KM Curve alone	Upregulated
Yang Y et al., 2018 [44]	China	June 2014 and August 2015	M-17/F-13	30	Tissue	qRT-PCR	NA	miR-122	NA	NA	NA	OS	KM Curve alone	Downregulated
Zhang S et al., 2019 [45]	China	NA	M-126/F-30	156	Tissue	Microarray analysis	NA	miR-142-3p, miR-150, miR-29b, and miR-29c	NKUC, NKDC, KSCC	N0-N3	T1, T2, T3 & T4	OS, DFS, DMFS, RFS	KM Curve alone	Downregulated
Wu L et al., 2019 [46]	China	March 2015 and November 2016	M-41/F-22	63	Saliva	Microarray analysis/ qRT-PCR	NA	miR-937-5p, miR-650, miR-3612, miR-4478, miR-4259, miR-3714, miR-4730, miR-1203, miR-30b-3p, miR-1321, miR-1202, and miR-575	NKDC	N0-N3	T1, T2, T3 & T4	NA	NA	Downregulated
Cui Z and Zhao Y, 2019 [47]	China	2002 and 2008	M-51/F-28	79	Tissue	RT-PCR	NA	miR-342-3p	NA	Studied but not mentioned exact stage	NA	OS	KM Curve alone	Downregulated
Huang Y et al., 2018 [48]	China	NA	M-41/F-21	62	Tissue	qRT-PCR	NA	miR-150	NKUC	NA	NA	OS	KM Curve alone	Upregulated
Feng X et al., 2018 [33]	China	August 2012 and July 2014	M-52/F-40	92	Tissue	qRT-PCR	NA	miR-495	KSCC	NA	NA	NA	NA	NA
Wan FZ et al., 2020 [49]	China	January 2013 to December 2015	M-58/F-12	72	Tissue	qRT-PCR	NA	miR-34c	NKUC, NKDC	N0-N3	T1, T2, T3 & T4	OS	KM Curve alone	Downregulated
Zhang Z et al., 2020 [50]	China	NA	M-39/F-9	48	Serum	qRT-PCR	Till May 2019	miR-29a, miR-26b, miR-29b, miR-143 and miR-125b	NKUC, NKDC	N0-N3	T1, T2, T3 & T4	PFS, OS	KM Curve alone	Downregulated
Huang Q et al., 2020 [51]	China	January 2016 to July 2019.	M-45/F-31	76	Tissue	qRT-PCR	NA	miR-192	NKUC, NKDC	N0-N3	T1, T2, T3 & T4	OS	KM Curve alone	Upregulated
Zhang H et al., 2020 [52]	China	2014 to 2016	M-270/F-181	389	Plasma	qRT-PCR	NA	miR-140-3p, miR-144-3p, miR-17-5p, miR-20a-5p, miR-20b-5p, and miR-205-5p	PDSC	Studied but not mentioned exact stage	T1, T2, T3 & T4	OS	KM Curve alone	miR-144-3p, miR-17-5p, miR-20a-5p, and miR-205-5p—Upregulated and miR-140-3p—Downregulated
Wang J et al., 2020 [53]	China	June 2013 and December 2016	M-102/F-48	150	Plasma	Microarray analysis/ qRT-PCR	Till December 2017	miR-214-3p	NA	NA	NA	RFS	KM Curve alone	Upregulated
Yang J et al. 2020 [54]	China	NA	M-78/F-71	149	Tissue	qRT-PCR	NA	miR-200c	NA	NA	NA	OS	KM Curve alone	Downregulated
Deng X et al. 2020 [55]	China	NA	M-75/F-35	110	Tissue	qRT-PCR	NA	miR-296-3p	NA	N0-N3	T1, T2, T3 & T4	OS	KM Curve alone	Downregulated
Lu T et al. 2020 [34]	China	July 2012 to March 2015	M-68/F-139	207	Tissue	qRT-PCR	NA	miR-BART13-3p and miR-BART7-3p	KSCC, NKUC, NKDC	N0-N3	T1, T2, T3 & T4	DMFS	KM Curve alone	NA

SCC—Squamous Cell Carcinoma (WHO type I); NKDC—Non-Keratinizing Differentiated Carcinoma (WHO type II); NKUC—Non-Keratinizing Undifferentiated Carcinoma (WHO type III); PDSCC—Partially differentiated Squamous Cell Carcinoma; NA: Not Available; M: Male; F: Female.

**Table 3:** The heterogeneity and hypothesis testing of the included studies in the meta-analysis.

		**Heterogeneity Testing and Hypothesis Testing**															
					**Classic Fail-Safe N**		**Orwin Fail-Safe N**		**Begg and Mazumdar Test**			**Dual and Tweedie (Random Effects)**					
	**Groups**	**Clinical Outcomes**			**Z Value**	***p*-Value**	**HR in Observed**		**Tau**	**Z Value**	***p*-Value**	**Observed**	**Q Value**		**Adjusted**	**Q Value**	
2018–2020	miRNAs in NPC	OS and PFS			6.34	0	1.08		0.07	0.45	0.65	1.589	130.34		1.08	193.44	
Combined Data (2013–2020)	miRNAs in NPC	OS and PFS			6.38	0	1.02		0.01	0.15	0.88	1.194	325.70		0.99	488.07	
		**Publication Bias**															
					**Fixed**			**Mixed/Random**				**Hypothesis Test**					
	**Groups**	**Heterogeneity**			**HR**	**95% CI**		**HR**	**95% CI**			**Fixed Effects Model**			**Random Effects Model**		
		**Q**	**P**	**I^2^**		**Low**	**High**		**Low**	**High**		**Z**	**P**	**Studies**	**Z**	**P**	**Studies**
2018–2020	miRNAs in NPC	130.34	0	84.66	1.08	1	1.16	1.59	1.25	2.02		2.01	0.04	21	3.82	0	21
Combined Data (2013–2020)	miRNAs in NPC	325.70	0	86.49	1.02	0.97	1.07	1.43	1.19	1.70		0.67	0.50	45	3.93	0	45

## Data Availability

Not applicable.

## Supplementary Materials

The following are available online at https://www.mdpi.com/article/10.3390/cancers13174369/s1, Supplementary Figure S1. Forest plot for survival parameter of miRNAs in NPC patients. Supplementary Figure S2. Funnel plot of Standard Error by Log hazard ratio correlating overall patient survival and microRNA expression. Supplementary Figure S3. Funnel plot with observed and imputed studies.
