# Supplementary material for: A Clinical Update on the Prognostic Effect of microRNA Biomarkers for Survival Outcome in Nasopharyngeal Carcinoma: A Systematic Review and Meta-Analysis
Source: Cancers (Basel). 2021 Aug 29;13(17):4369. doi: 10.3390/cancers13174369 (PMC8431423; doi:10.3390/cancers13174369)
Supplement: Supplementary file 1 [file cancers-13-04369-s001.zip › cancers-1313947-supplementary.pdf]

Supplementary Figures

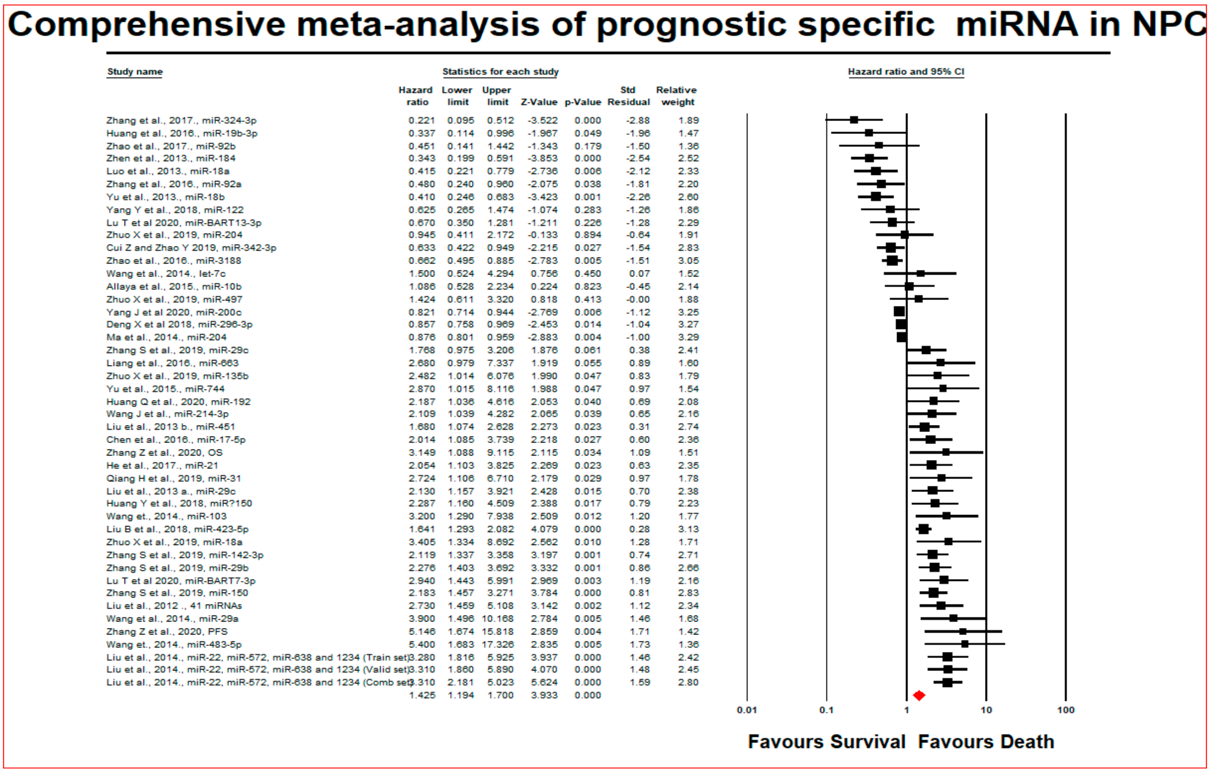

Supplementary Figure S1. Forest plot for survival outcome of miRNAs in NPC patients. Meta-analysis of prognostic specific miRNAs in NPC. Pooled HRs for NPC patients' survival data were analysed using Comprehensive Meta-Analysis (CMA) software (version 3.3.070). Impact of HR value on NPC patients: raw HR data with 95% CIs from individual studies are graphically represented in the plot. A meta-analysis statistically combines HRs from all studies and gives a more precise estimate of the effect. In the forest plot, the red diamond graphically represents the pooled effect estimate of survival for NPC patients randomly assigned to miRNA evaluation. The black box and line indicate the effect size of miRNA expression in the included studies with a 95% CI. An HR of 1 suggests no difference in risk of NPC patients' survival (line of no effect or difference). An HR> 1 indicates an increased risk of patients' survival whereas a HR< 1 suggests a reduced risk of patients' survival. In our study, the miRNAs increased the likelihood of NPC patients' death by 42.3%.

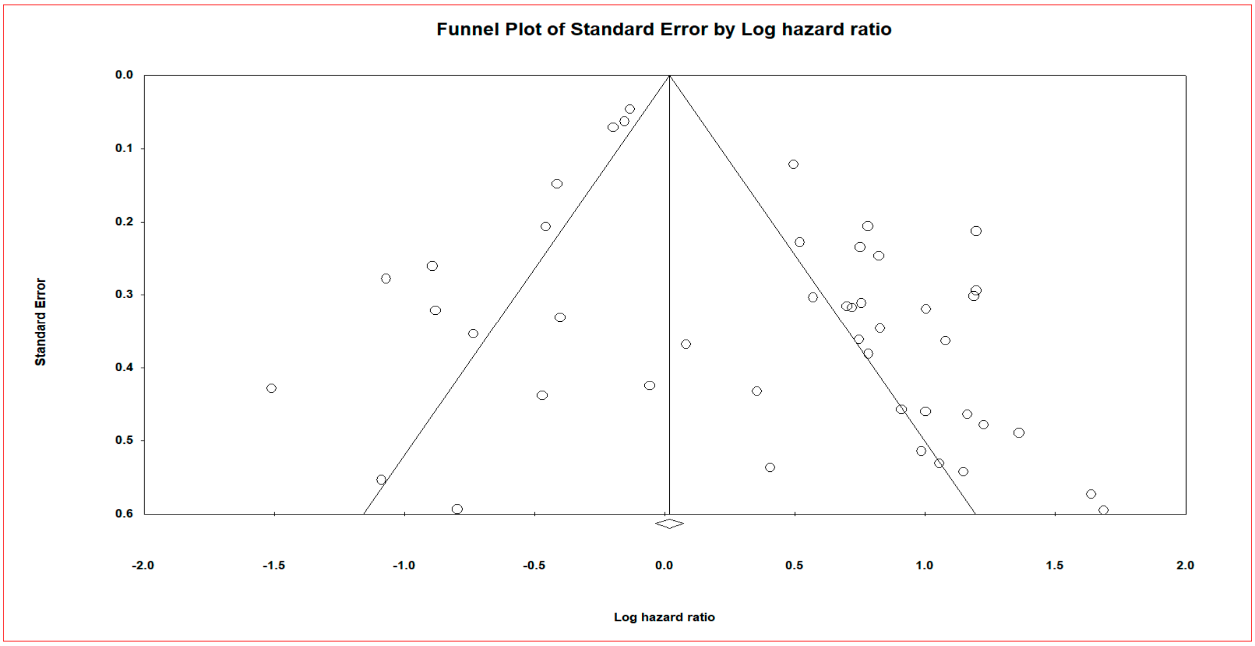

Supplementary Figure S2. Funnel plot of Standard Error by Log hazard ratio correlating overall patient survival and microRNA expression. The funnel plot measures the study size standard error and precision on the vertical axis as a function of effect size on the horizontal axis. Dots represent the individual study and most of this area contains regions of high significance revealing that publication bias would be represented in the form of asymmetry. This would reflect the fact that smaller studies (which appear toward the bottom) are more likely to be published if they have larger than average effects, which makes them more likely to meet the criterion for statistical significance.

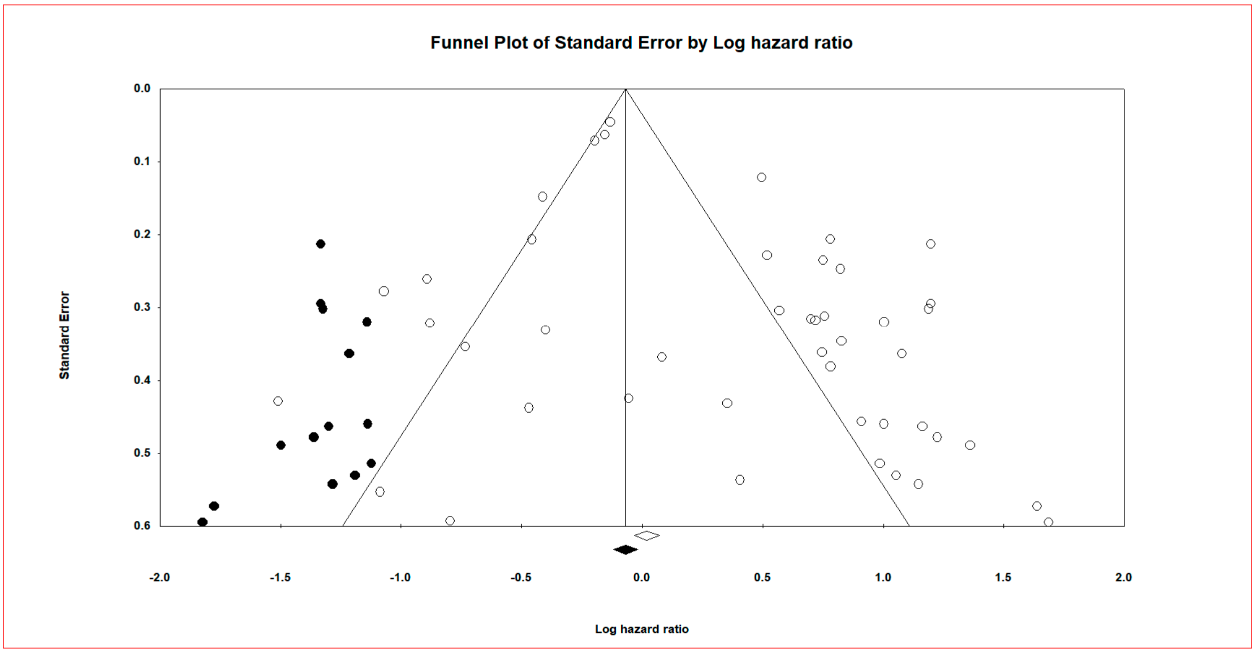

Supplementary Figure S3. Funnel plot with observed and imputed studies. The funnel plot measures the study size standard error and precision on the vertical axis as a function of effect size on the horizontal axis. Dots represent the individual study and most of this area contains regions of high significance, which reveals that publication bias would be represented in the form of asymmetry. This would reflect the fact that smaller studies (which appear toward the bottom) are more likely to be published if they have larger than average effects, which makes them more likely to meet the criterion for statistical significance.
